# Clinical and hemodynamic outcomes of the Perceval sutureless aortic valve from a real-world registry

**DOI:** 10.1093/icvts/ivad103

**Published:** 2023-06-12

**Authors:** Giovanni Concistré, Max Baghai, Giuseppe Santarpino, Alistair Royse, Maximilian Scherner, Giovanni Troise, Mattia Glauber, Marco Solinas

**Affiliations:** Department of Adult Cardiac Surgery, G Pasquinucci Heart Hospital, Massa, Italy; Cardiothoracic Surgery, King's College Hospital NHS Foundation Trust, London, UK; Department of Cardiac Surgery, Città di Lecce Hospital, GVM Care & Research, Lecce, Italy; Cardiothoracic Surgery, The Royal Melbourne Hospital, Parkville, VIC, Australia; Department of Cardiothoracic Surgery, University of Magdeburg, Magdeburg, Germany; Department of Cardiovascular Surgery, Poliambulanza Foundation/Cardiac Surgery Unit, Brescia, Italy; Department of Cardiac Surgery, Gruppo San Donato, Milano, Italy; Department of Adult Cardiac Surgery, G Pasquinucci Heart Hospital, Massa, Italy

**Keywords:** Aortic stenosis, Aortic valve replacement, Sutureless valves, Real-world evidence

## Abstract

**OBJECTIVES:**

Perceval sutureless valve has been in clinical use for >15 years. The aim of this study is to report the real-word clinical and haemodynamic performance from the SURE-aortic valve replacement international prospective registry in patients who underwent aortic valve replacement with Perceval valve.

**METHODS:**

From 2011 to 2021, patients from 55 institutions received a Perceval valve. Postoperative, follow-up, and echocardiographic outcomes were analysed.

**RESULTS:**

A total of 1652 patients were included; mean age was 75.3 ± 7.0 years (53.9% female); mean EuroSCORE II was 4.1 ± 6.3. Minimally invasive approach was performed in 45.3% of patients; concomitant procedures were done in 35.9% of cases. Within 30 days, 0.3 and 0.7% valve-related reinterventions were reported. Transient ischaemic attack, disabling and non-disabling strokes were limited (0.4%, 0.4% and 0.7%, respectively). Pacemaker implant was required in 5.7% of patients. Intra-prosthetic regurgitation ≥2 was present in 0.2% of cases, while paravalvular leak ≥2 in only 0.1%. At a maximum follow-up of 8 years, 1.9% of cardiovascular deaths and 0.8% of valve-related reintervention occurred. Among the 10 cases of structural valve deterioration (mean 5.6 ± 1.4 years after implant; range: 2.6–7.3 years), 9 were treated with a transcatheter vale-in-valve implantation and 1 with explant. Mean pressure gradient decreased from 45.8 ± 16.5 mmHg preoperatively to 13.3 ± 5.2 mmHg at discharge and remained stable during follow-up.

**CONCLUSIONS:**

This experience represents the largest prospective real-world cohort of patients treated with Perceval showing that Perceval is a safe and effective alternative to conventional surgical aortic valve replacement, providing favourable clinical and haemodynamic results also at mid-term follow-up.

**Figure:**
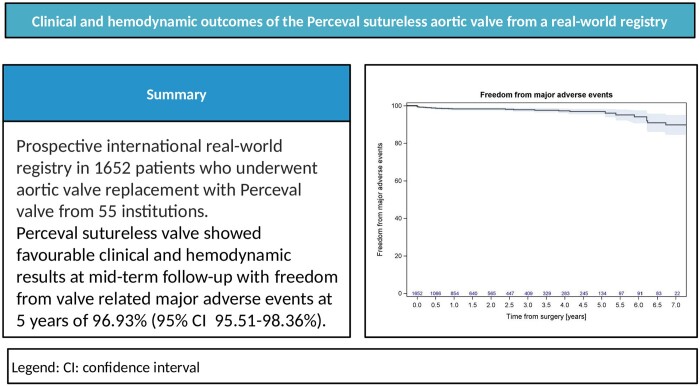

## INTRODUCTION

Open-heart operations with aortic valve replacement (AVR) remain the standard treatment for patients with severe symptomatic aortic valve disease [1, 2]. According to the Society of Thoracic Surgeons database, the operative risk of AVR has dramatically improved in the last decade, showing a reduction of mortality from 4.3% to 2.6% [3, 4]. Despite these results, elderly patients and patients with significant comorbidities referred for AVR are still at high risk for surgery.

The concept of the sutureless aortic valve prosthesis was initially reported by Magovern and Cromie in the early 60s [5] to facilitate the replacement of the aortic valve, shorten operating time and avoid the complications of prolonged cardiopulmonary bypass (CPB) as well as aortic cross-clamp (ACC) time. However, this innovative idea was abandoned because of valve-related morbidity, such as frequent perivalvular leaks, dehiscence in patients with large dilated aortic roots and thromboembolic complications [6, 7]. In recent years, sutureless aortic valve implantation has gained interest because of the rapid development of new valve technologies. The Perceval valve (Corcym S.r.l., Saluggia, Italy) is a self-expandable sutureless aortic bioprosthesis and several reports have shown promising results in terms of mortality, morbidities and haemodynamic performances [8–11]. The aim of this study is to report the real-word clinical and haemodynamic performance from the SURE-AVR (Sorin Universal REgistry on Aortic Valve Replacement) registry in patients who underwent AVR with the Perceval valve.

## PATIENTS AND METHODS

### Ethical statement

This study was approved by the Ethics Committees/Institutional Review Board and/or health authorities were performed according to local regulations. All patients enrolled in the study provided written informed consent.

### Study design

The SURE-AVR registry (NCT02679404), sponsored by Corcym S.r.l., was a prospective observational registry conducted at 73 sites in 18 countries in Europe, Canada, USA and Australia; patients treated with any of the commercially available Corcym aortic products were eligible for enrolment. This paper reports the final analysis of the Perceval subgroup.

Preoperative, periprocedural, follow-up clinical and echocardiographic parameters, as well as clinical outcomes, were analysed for all patients.

The registry was conducted according to the International Conference on Harmonization guidelines, Good Clinical Practice and local regulations. Ethics committee and/or institutional review board approval was obtained as required by local regulations. All patients gave informed consent to participate.

Patients were enrolled in a sequential and prospective manner and were treated based on the standard of care at participating sites.

Baseline data were entered into an electronic case report form by trained study coordinators, and included demographic, clinical, echocardiographic and surgical data. Follow-up visits were performed according to the centres’ usual practices (by telephone call, referring physician or clinical visit) at 1 year and annually up to 5 years, with follow-up at 7 years in selected centres.

No specific inclusion and exclusion criteria other than the indications and contraindications specified in the ‘instructions for use’ of the Perceval valve were implemented, as the aim of the study was to report on the standard of care at participating centres. The main contraindications for the use of the Perceval valve are the aneurysmal dilation or dissection of the ascending aortic wall, known hypersensitivity to nickel alloys and presence of anatomical characteristics indicating enlargement of aortic root. The primary end-point was to evaluate the 5-year freedom from site reported valve-related major adverse events (MAE), defined as death, stroke and/or reintervention (involving surgery or any other invasive therapy), while secondary end-points were site reported adverse events and haemodynamic results through the 5-year follow-up.

### Study device

The Perceval valve is a self-anchoring, self-expanding, sutureless, surgical aortic bioprosthesis indicated for the replacement of damaged or malfunctioning native aortic heart valves or prostheses. This bioprosthesis has a functional component, comprising bovine pericardium, stabilized in a buffered glutaraldehyde solution and a super-elastic Nitinol stent, which has the dual role of supporting the valve and anchoring it to the aortic root with no permanent sutures. The valve is stored in an aldehyde-free solution, and no rinse is required before implantation. The Perceval Plus model features an innovative tissue treatment (FREE) that addresses both sources of tissue mineralization (phospholipids and aldehydes) [12]. Prior to implantation, the prosthesis diameter is reduced to a suitable size for loading it onto a delivery system. The valve is then positioned and released in the aortic root under direct visualization and subsequently post-dilated using a dedicated balloon catheter. The device is available in 4 sizes (Small, Medium, Large, Extra-large) covering annular diameters ranging from 19 to 27 mm. Implantation can be performed using a traditional surgical approach, or through minimally invasive cardiac surgery procedures for which the sutureless design is particularly suited.

### Clinical outcomes

Clinical success was defined as a successful valve implantation without the occurrence of major adverse events by the time of hospital discharge.

Investigator-reported major adverse events were defined as death (all-cause, cardiovascular, non-cardiovascular), stroke and reintervention (surgery or any other cardiac invasive therapy). Serious valve-related adverse events included bleeding, thromboembolism, valve thrombosis, endocarditis, non-structural dysfunction and structural valve deterioration, according to the VARC-2 definitions [13].

Severity of valve regurgitation was classified as mild (grade 1+), moderate (grade 2+), moderate to severe (grade 3+) or severe (grade 4+) [14]. Echocardiographic and haemodynamic data were collected. Early outcomes were defined as those occurring up to 30 days after the procedure while late outcomes as those occurring >30 days after procedure.

### Statistical analysis

Variables are described as mean ± standard deviation or median (quartile Q1, Q3; range) for continuous variables, and as number (%) for categorical variables. Outcomes are reported as descriptive statistics. The proportions of early adverse events were calculated as the total number of events divided by the total number of patients. Linearized late complication rates (and 95% confidence intervals) were calculated as the number of late events (>30 days) divided by the number of late patient-years. Survival and freedom from events were evaluated using the method of Kaplan–Meier, with 95% confidence interval (CI) around the estimates. The statistical analyses were performed using SAS^®^ (Release 9.4, by SAS Institute Inc., Cary, NC, USA).

## RESULTS

Between March 2011 and June 2021, 1652 patients underwent AVR with the Perceval sutureless valve in 55 International institutions. The characteristics of the study population are detailed in Table 1. There were 891 (53.9%) female patients and overall mean age was 75.3 (7.0) years with a mean EuroSCORE II of 4.1 (6.3). The indication for the sutureless implant was stenosis in 74.6% (1233), steno-regurgitation in 18.2% (300) and aortic regurgitation in 5.4% (89) of the patients. Most of the patients (1361, 82.4%) were in New York Heart Association class II or III; 76 (4.6%) were in class IV.

**Table 1:** Baseline clinical characteristics

Baseline characteristics	*n* = 1652
Age (years), mean (SD)	75.3 (7.0)
Female, *n* (%)	891 (53.9)
Dyslipidaemia, *n* (%)	937 (56.7)
Diabetes, *n* (%)	514 (31.1)
Chronic lung disease, *n* (%)	228 (13.8)
Renal insufficiency, *n* (%)	184 (11.1)
Peripheral vascular disease, *n* (%)	103 (8.5)
Previous cardiac procedures	270 (16.3)
Previous myocardial infarction, *n* (%)	162 (9.8)
Previous CVA, *n* (%)	107 (6.5)
Left ventricular ejection fraction, *n* (%)	57.0 (11.0)
NYHA class II–III, *n* (%)	1361 (82.4)
Preop sinus rhythm, *n* (%)	1290 (78.1)
Endocarditis, *n* (%)	35 (2.1)
Bicuspid valve, *n* (%)	132 (8.0)
EuroSCORE II (%), mean (SD)	4.1 (6.3)

SD: standard deviation; CVA: cerebrovascular accidents; NYHA: New York Heart Association.

The majority of patients (1290, 78.1%) were in sinus rhythm and 205 (12.4%) in atrial fibrillation. Overall, 16.3% (270) of the patients had undergone cardiac intervention before the sutureless implant.

### Surgical procedures

Operative data are reported in Table 2. Almost half (744, 45.3%) of patients underwent a minimally invasive approach (718, 67.8% in isolated AVR) and a concomitant procedure was reported in 35.9% (593) of patients. In most cases (426, 25.8%), a coronary artery bypass graft was performed, while a mitral or tricuspid valve procedure was done in 5.8% (95) and 2.8% (47) of patients, respectively.

**Table 2:** Operative data

Operative data	*N* = 1652
Approach	
Sternotomy, *n* (%)	899 (54.4)
Mini-sternotomy, *n* (%)	420 (25.4)
Mini-thoracotomy, *n* (%)	324 (19.6)
Missing, *n* (%)	9 (0.5)
Minimally invasive approach in isolated AVR, *n* (%)	718 (67.8)
First successful implant, *n* (%)	1628 (98.5)
Perceval size, *n* (%)	
Small	241 (14.6)
Medium	537 (32.5)
Large	594 (36.0)
Extra large	280 (16.9)
Concomitant procedure, *n* (%)	593 (35.9)
ICU stay (days) (median) (IQR)	2.0 (1.0–3.0)
Total length of stay (median) (IQR)	9.0 (7.0–13.0)
Cross clamp time (min)—overall, mean (SD)	61.0 (29.9)
Pump time (min)—overall, mean (SD)	90.3 (42.2)
Cross clamp time (min)—isolated AVR, mean (SD)	51.0 (20.5)
Pump time (min)—isolated AVR, mean (SD)	77.4 (30.8)
Cross clamp time (min)—isolated AVR minimally invasive approach, mean (SD)	51.9 (20.0)
Pump time (min)—isolated AVR, minimally invasive approach mean (SD)	79.7 (30.4)
Cross clamp time (min)—concomitant procedures, mean (SD)	79.1 (35.2)
Pump time (min)—concomitant procedures, mean (SD)	113.3 (49.4)

AVR: aortic valve replacement; ICU: intensive care unit; IQR: interquartile range; SD: standard deviation.

The Perceval valve was successfully implanted on first attempt in 1628 (98.5%) patients. In the remaining 24 patients, a new sutureless valve was implanted, due to initial malpositioning of bioprosthesis. The implanted valve size was Large (L) in 36% (594) of patients, Medium (M) in 32.5% (537), Extra-large (XL) in 16.9% (280) and Small (S) in 14.6% (241).

Mean overall ACC time was 61.0 (29.9) min and CPB time was 77.4 (30.8) min. Operative times for isolated and combined procedures are reported in Table 2. Median intensive care unit and total length of stay were 2.0 (1.0–3.0) and 9.0 (7.0–13.0) days.

### Early and late outcomes

Early and late outcomes are detailed in Table 3. Five cardiovascular deaths (0.3%) were reported in the early period, 4 due to heart failure and 1 to myocardial infarction, while there were 8 (0.5%) non-cardiovascular death. Among the 11 cases (0.7%) of valve-related reinterventions, 7 were due to non-structural valve disfunction (1 case treated with transcatheter aortic valve replacement (TAVR) valve-in-valve on day 6 after the procedure, 3 explants on days 0, 9 and 11 post operative and 3 surgeries without explant all intraoperative), 2 were due to a valve malpositioning (treated with device explant), 1 case due to a dislodgement (successfully repositioned on the same day of surgery) and 1 case was related to a valve explant (incomplete expansion of the stent valve caused by the calcification of the aortic root).

**Table 3:** Early (≤30 days) and late (>30 days) outcomes

	Early outcomes	Late outcomes
	*N* (% AEs in 1652 patients)	*N* (% in 2848.1 late pt-yrs) (2-side 95% CI)
All deaths	13 (0.8)	127 (4.5) (3.7–5.3)
Cardiovascular deaths	5 (0.3)	55 (1.9) (1.5–2.5)
Non-cardiovascular deaths	8 (0.5)	70 (2.5) (1.9–3.1)
Valve-related reintervention	11 (0.7)	23 (0.8) (0.5–1.2)
SVD	0	6 (0.2) (0.1–0.4)
TIA	7 (0.4)	13 (0.5) (0.3–0.8)
Disabling stroke	7 (0.4)	7 (0.2) (0.1–0.5)
Non-disabling stroke	12 (0.7)	12 (0.4) (0.2–0.7)
Thromboembolism	2 (0.1)	3 (0.1) (0.0–0.3)
Bleeding	22 (1.3)	17 (0.6) (0.4–0.9)
Intra-prosthetic regurgitation (≥2)	3 (0.2)	13 (0.5) (0.3–0.8)
PVL (≥2)	2 (0.1)	0 (0)
Intra and PVL (≥2)	0 (0)	0 (0)
Endocarditis	0 (0)	14 (0.5) (0.3–0.8)
Valve thrombosis	0 (0)	0 (0)
Myocardial infarction	3 (0.2)	13 (0.5) (0.3–0.8)
Pacemaker implant	95 (5.7)	48 (1.7) (1.3–2.2)

CI: confidence interval; PVL: paravalvular leak; SVD: structural valve deterioration; TIA: transient ischaemic attack; AEs: adverse events.

Transient ischaemic attack, disabling and non-disabling strokes were reported in 0.4% (7), 0.4% (7) and 0.7% (12) of patients. Bleeding occurred in 1.3% (22) of cases.

Intra-prosthetic regurgitation ≥2 was present in 0.2% (3) of the cases, while paravalvular leak ≥2 in only 0.1% (2). No case of intra-prosthetic regurgitation ≥2 associated to paravalvular leak ≥2. A permanent pacemaker implant was required in 5.7% (95) of patients.

Median study follow-up duration was 12.2 (0.4–36) months (maximum of 8 years), with a cumulative follow-up of 2848.1 late patient-years. The 5-year follow-up visit was done for the 87.3% of the expected visits (206 patients out of the 236 reaching the follow-up window).

In the late period, 55 cardiovascular (1.9%/pt-yrs) and 127 non-cardiovascular (4.5%/pt-yrs) deaths were reported. Twenty-three (0.8%/pt-yrs) cases of valve-related reintervention were registered due to endocarditis (7 cases), structural valve deterioration (10 cases) and non-structural valve dysfunction (6 cases). Among the 10 cases of reintervention due to structural valve deterioration [mean 5.6 (1.4) years after implant; range: 2.6–7.3 years], 9 were treated with a TAVR valve-in-valve implantation and 1 valve was explanted. Among the 6 cases of non-structural valve dysfunction, 3 were treated with TAVR valve-in-valve implantation, while in 3 patients, an explant was required.

Transient ischaemic attack occurred in 13 patients (0.5%/pt-yrs), while disabling and non-disabling strokes in 7 (0.2%/pt-yrs) and 12 (0.4%/pt-yrs), respectively. Intra-prosthetic regurgitation ≥2 was present in 15 patients (0.5%/pt-yrs), while no case of paravalvular leak ≥2 was recorded. Permanent pacemaker implant was required in 48 (1.7%/pt-yrs) cases.

**Figure 1:**
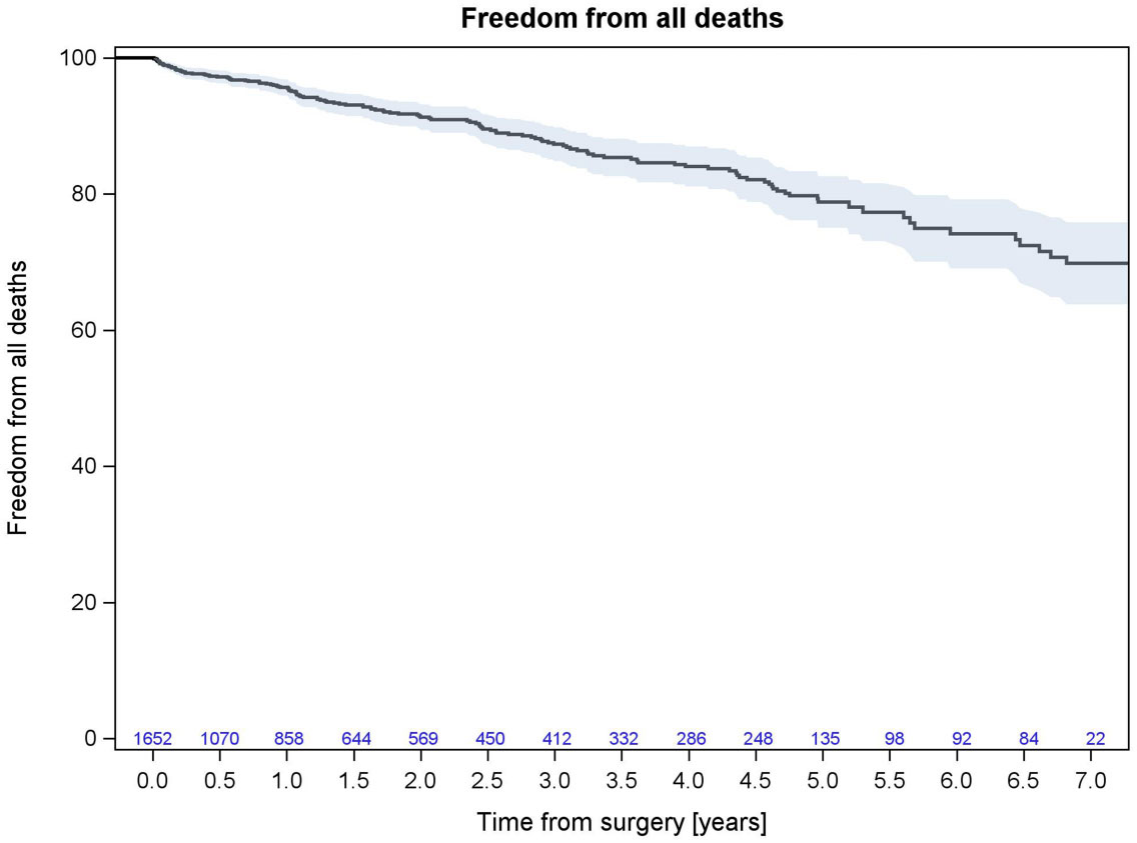
Kaplan–Meier curve for overall survival.

**Figure 2:**
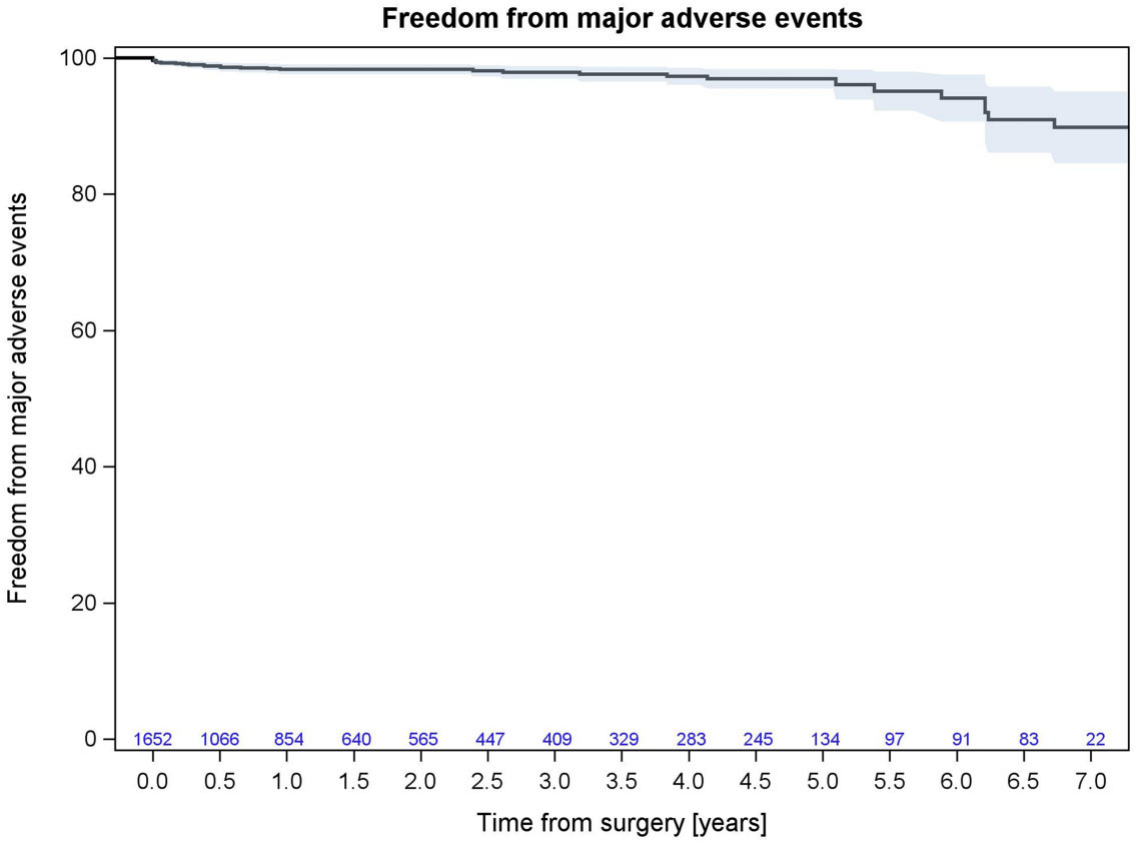
Kaplan–Meier curve for valve-related major adverse events (death, reintervention, stroke, and/or transient ischaemic attack).

Survival at 5-year follow-up was 78.82% (95% CI: 75.03–82.61%; Fig. 1), while freedom from valve-related major adverse events (death, reintervention, stroke and/or TIA) at 5 years was 96.93% (95% CI: 95.51–98.36%; Fig. 2).

### Echo data

As per study protocol, the echocardiographic data were collected per hospital practice. The mean echocardiographic follow-up was 549.9 (759.8) days. Echocardiographic data were available for 1228 patients at discharge, 512 at 1 year and 197 at 5 years (74.7%, 66.7% and 95.6% of the patients reaching the follow-up window, respectively).

Mean aortic pressure gradient decreased from 45.8 (16.5) mmHg preoperatively to 13.3 (5.2) mmHg at discharge and peak pressure gradient from 74.7 (25.9) mmHg to 24.7 (10.5) mmHg. Mean effective orifice area was 0.8 (0.3) cm^2^ before surgery and increased up to 1.7 (0.6) at discharge. All values remained stable up to 5 years follow-up (Table 4).

**Table 4:** Echocardiographic data

Echo data	Preoperative	Discharge	1 year	5 years
	*n* = 1608	*n* = 1228	*n* = 512	*n* = 197
Effective orifice area (cm^2^), mean (SD)	0.8 (0.3)	1.7 (0.6)	1.7 (0.5)	1.5 (0.4)
Mean gradient (mmHg), mean (SD)	45.8 (16.5)	13.3 (5.2)	11.9 (5.5)	13.7 (10.0)
Peak gradient (mmHg), mean (SD)	74.7 (25.9)	24.7 (10.5)	20.5 (8.9)	23.0 (15.0)

SD: standard deviation.

## DISCUSSION

This study shows clinical and echocardiographic results with the Perceval sutureless bioprosthesis in 1652 patients undergoing AVR, included in SURE-AVR registry. Our data demonstrate that the implantation of Perceval bioprosthesis is a safe and feasible procedure associated with low mortality and excellent haemodynamic performance at 5-years follow-up. Unlike previous reports, this study was prospective and analysed the largest multicentre cohort of patients implanted with a Perceval bioprosthesis.

Sutureless bioprosthesis represents an innovative approach for surgical AVR and has been designed to allow faster implantation, reducing CPB and ACC time. This is an advantage for all patients, regardless of the risk profile. Therefore, sutureless aortic valve implantation might be an alternative treatment option for patients at high-risk for mortality and morbidity after open-heart surgery. First clinical results of Perceval bioprosthesis were reported in 2011 by the group of Flameng and colleagues [15]. Fischlein *et al.* reported low 1-year event rates in intermediate-risk patients undergoing AVR from a large multicentre cohort study [8].

In our experience, 30-day cardiovascular death was 0.3% (5/1652) and survival was 78.8% at 5 years follow-up. Early outcomes showed a low rate of neurological events and reinterventions. At 30-days, 7 patients had a non-structural valve disfunction, 2 a valve malpositioning and 1 case was related to a valve explant due to incomplete expansion of the stent valve caused by the calcification of the aortic root. Median of intensive care unit stay was 2.0 days and total length of stay 9.0 days. At discharge, mean transvalvular gradient was 13.3 (5.2) mmHg. Compared to other series [11, 18–20], we found similar results in terms of paravalvular leakage and haemodynamic performance. In our opinion, oversizing the prosthesis will not reduce the incidence of leakage; conversely, it might lead to incomplete expansion of the bioprosthesis with infolding of the annular portion. Sizing of the device is important as the Perceval is designed to expand to an outer diameter larger than the patient’s measured annular diameter. The expansion of the stent provides the proper radial force to secure the Perceval in place for stability at physiological pressure, flow and movement. The Perceval bioprosthesis selected for implant should match the measured diameter of the aortic annulus. Margaryan *et al.* analysed 54 patients implanted with the Perceval who had preoperative contrast-enhanced multidetector-row computed tomography (MDCT). Echocardiographic measurements showed lower accuracy compared to MDCT measurements. They concluded that for precise aortic annulus measurement, contrast-enhanced MDCT is preferable [16]. In a study by Massa Center, Cerillo *et al.* investigated the relationship between a computed tomography measure of the degree of oversizing and the early haemodynamic and clinical outcomes in patients undergoing AVR with Perceval valve. The degree of oversizing of the implanted prosthesis was calculated as the ratio between the patients' aortic annulus cross-sectional area and the *ex vivo* cross-sectional area of the implanted prosthesis in 151 Perceval patients who underwent preoperative cardiac computed tomography. This value was then entered in a multivariate analysis to ascertain its role as a predictor of increased postoperative gradient. The degree of oversizing of the implanted prosthesis was the most important predictor of increased postoperative gradient (odds ratio: 1.264; 95% confidence interval: 1.147–1.394; *P* < 0.0001). Interestingly, other relevant factors (patients’ body surface area, prosthesis size) were not associated with increased gradients. This study demonstrates that excessive oversizing should be avoided in Perceval patients and suggests that a different sizing algorithm, possibly based on cardiac computed tomography, should be developed [17]. Based on this study, since 2017, many Institutions of our registry have changed their sizing procedure avoiding oversizing and basing the choice of bioprosthesis size on CT data, confirmed with intraoperative sizing. A recently published series from Szecel *et al.*, comparing the results of Perceval obtained before and after the change in sizing procedure, showed a decrease of pacemaker rate and improved haemodynamics with the new procedure, confirming that avoiding oversizing is crucial in obtaining the best haemodynamic and clinical outcomes with the Perceval sutureless valve [18]. We look forward to the long-term results of implanted prostheses after the sizing procedure change.

The reduced time needed for implantation is a potential advantage of this prosthesis. In our study, overall cross-clamp time was 61.0 (29.9) min and pump time 90.3 (42.2) min. In Society of Thoracic Surgeons database, the times of ACC and CPB times for AVR in full sternotomy are 77.9 and 106.4, respectively. In 67.8% of patients with isolated AVR, Perceval was implanted in minimally invasive approach. A meta-analytical study showed CPB time of 104.4 min for the minimal access group that underwent AVR versus 94.0 min for the conventional access group (*P* < 0.00001) [19]. Our experience showed CPB time of 79.7 (30.4) min for the minimal access group that underwent AVR with Perceval valve. As minimally invasive AVR has shown longer CPB and ACC time than conventional surgery, we strongly believe that sutureless technology might be the solution for less invasive approaches. A limitation is that we do not have the implantation time from the moment the aortic valve is excised to the moment the new valve is fully expanded. The advantages of Perceval implantation in ministernotomy approach have been described by Fischlein *et al.* [9]. On the other hand, we reported the advantages of AVR with sutureless implantation through a right minithoracotomy [20]. Besides the possible facilitation of minimally invasive approach, this prosthesis may also reduce CPB and ACC times in associated procedures. In our experience, there was a significant number of patients who had concomitant bypass surgery (25.8%), mitral (5.8%) and tricuspid (2.8%) procedures. Mean transvalvular gradient at 5 years follow-up was 13.7 (10.0) mmHg. The rate of perioperative pacemaker implantation was 5.7%. This result is the same of the largest European multicentre experience [21]. At 5 years follow-up, freedom from valve-related major adverse events was 95.11%. Seven patients were reoperated for endocarditis, while 6 patients had non-structural valve disease. Structural Perceval degeneration requiring reintervention occurred in 10 patients at mean years after first implantation of 5.6 (1.4) (range: 2.6–7.3 years). Nine of these patients underwent transcatheter valve-in-valve implantation, with a safe, feasible and favourable procedure, thanks to the Perceval stent design that allows for clear landmarks visibility and coronary ostia patency. The remaining patient underwent surgical sutured valve replacement. Durability of sutureless bioprosthesis at 5 years is comparable with sutured bioprosthesis, despite in the first phase of our experience (2011–2016), we often oversized the prosthesis. In a study of Johnston, actuarial estimates of explant of Carpentier-Edwards PERIMOUNT stented bovine pericardial prostheses for structural valve degeneration at 10 and 20 years were 1.9% and 15% overall [22]. In our 5 years’ experience, the rate of Perceval degeneration is 0.4%. Perceval Plus was implanted in 233 patients. This is a new generation of Perceval with a new tissue treatment, which combines an adequate phospholipid reduction and aldehyde neutralization with storage in an aldehyde-free solution. This combination enhances the anticalcification properties and may thereby improve long-term durability of the tissue. Furthermore, the protrusion in the left ventricle of the new valve stent has been reduced and this should decrease the incidence of blocks and pacemaker implantation.

### Limitations

This study has the limitations of any observational registry involving no monitoring and no adjudication of patient inclusion and adverse events, with no core laboratory to review images. It is a prospective non-randomized study; therefore, it lacks a comparative arm. Since follow-up visits were performed according to the site’s routine practice, the echocardiographic follow-up is not available for all the patients; moreover, the follow-up was not systematic nor similar across the centres. However, the SURE-AVR is the largest multicentric prospective registry on the Perceval valve in real-world patient population.

## CONCLUSIONS

Our multicentre, real-world experience with Perceval sutureless valve showed favourable clinical and haemodynamic results at mid-term follow-up. Sutureless technology and its future evolutions, associated with minimally invasive approach, might be considered an alternative treatment option for AVR, especially in high-risk patients.

## Data Availability

The data underlying this article will be shared on reasonable request to the corresponding author.

## Notes

Presented at the 36th EACTS Annual Meeting, 6 October 2022, Milan, Italy.

## ABBREVIATIONS

ACC: Aortic cross-clamp
AVR: Aortic valve replacement
CPB: Cardiopulmonary bypass
MDCT: Multidetector-row computed tomography
TAVR: Transcatheter aortic valve replacement
